# Role of gut microbiota and bacterial metabolites in mucins of colorectal cancer

**DOI:** 10.3389/fcimb.2023.1119992

**Published:** 2023-05-17

**Authors:** Ming Gu, Weixiang Yin, Jiaming Zhang, Junfeng Yin, Xiaofei Tang, Jie Ling, Zhijie Tang, Weijuan Yin, Xiangjun Wang, Qing Ni, Yunxiang Zhu, Tuo Chen

**Affiliations:** Department of General Surgery, Affiliated Hospital of Yangzhou University, Yangzhou, China

**Keywords:** mucins, gut microbiota, bacterial metabolites, bacteria-related therapies, colorectal cancer

## Abstract

Colorectal cancer (CRC) is a major health burden, accounting for approximately 10% of all new cancer cases worldwide. Accumulating evidence suggests that the crosstalk between the host mucins and gut microbiota is associated with the occurrence and development of CRC. Mucins secreted by goblet cells not only protect the intestinal epithelium from microorganisms and invading pathogens but also provide a habitat for commensal bacteria. Conversely, gut dysbiosis results in the dysfunction of mucins, allowing other commensals and their metabolites to pass through the intestinal epithelium, potentially triggering host responses and the subsequent progression of CRC. In this review, we summarize how gut microbiota and bacterial metabolites regulate the function and expression of mucin in CRC and novel treatment strategies for CRC.

## 1 Introduction

Globally, colorectal cancer (CRC) has become a major health burden due to its higher incidence and mortality. Epidemiological data indicate that CRC ranks third in incidence with 1.9 million new cases and is the second most common cause of cancer mortality (Sung et al., 2021). In China, over 300,000 new cases and 191,000 deaths are reported annually (Chen et al., 2016; Chen et al., 2018b). As with many diseases, the etiology of CRC is multi-factors involving genetic and environmental factors (Song and Chan, 2019). While genetic susceptibility implicated in CRC is well-described, the incidence of CRC in genetic predisposition syndromes, including familial adenomatous polyposis, Peutz–Jeghers syndrome, and Lynch syndrome, only accounts for a minority of CRC cases (Boland et al., 2018; Hryhorowicz et al., 2022). Thus, it is suggested that environmental factors play a major role in the initiation and progression of CRC (Keum and Giovannucci, 2019). Among environmental factors, the gut microbiome has been increasingly considered a modulator of CRC (Zou et al., 2018).

The community of bacteria, fungi, archaea, phages, and protists is referred to as the microbiota. These microorganisms within the gastrointestinal tract are named “gut microbiota.” There are approximately 10^13^ to 10^14^ bacteria living in the gut, which contain 10 times more than human cells and outnumber human genes by a factor of 100 (Sears, 2005; Wardman et al., 2022). These microorganisms play an important role in maintaining the intestinal epithelium (Hill et al., 2017), harvesting energy (Vandeputte, 2020), and maturing immunity (Shi et al., 2017). Meanwhile, the shift in their composition has been associated with cardiovascular diseases (Brown and Hazen, 2018), metabolic diseases (Maruvada et al., 2017), and digestive diseases (such as inflammatory bowel disease and CRC) (Kostic et al., 2014; O'Keefe, 2016). Accumulating evidence shows that the initiation of CRC is triggered by the dysfunction of colonic mucosal barrier colonization by specific gut microbiota (Sheng et al., 2012; Wong and Yu, 2019). These bacteria cause changes in the tumor microenvironment, allowing for colonization by opportunistic bacteria that facilitate disease progression.

**Figure 1:**
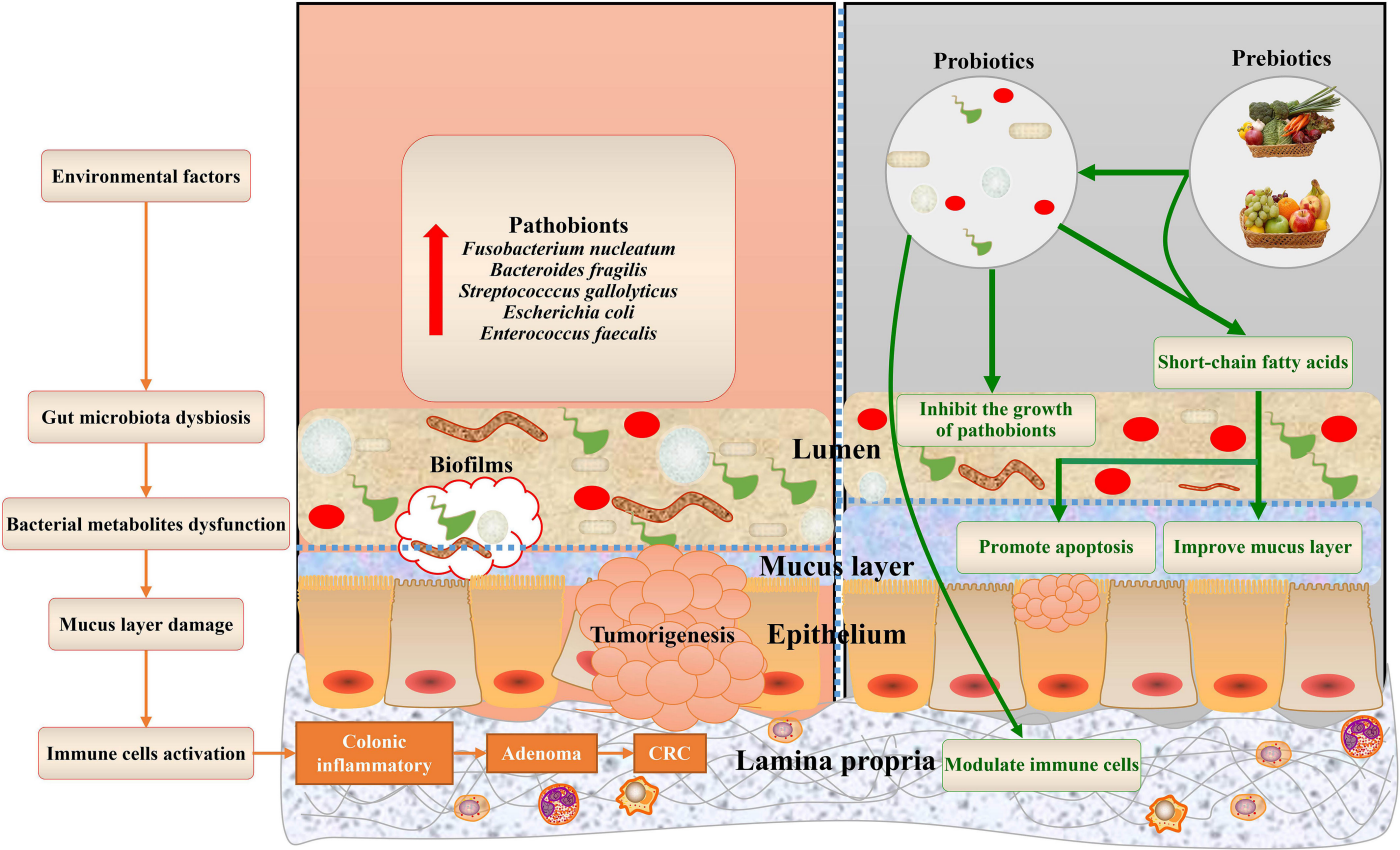
Gut microbiota bacterial metabolites associated with mucins of colorectal cancer, and potential bacteria-related therapies. The dysbiosis of the gut microbiota and dysfunctions of bacterial metabolites, aggravated by environmental factors, contribute to mucus layer damage during colorectal cancer. This schematic also summarizes the health benefits of prebiotics and probiotics that cause alterations to mucins of CRC.

The mucus layer acts as the first gatekeeper against environmental and microbial insults. Among the components of the mucus layer, mucins, mainly secreted by goblet cells, are found throughout the gastrointestinal epithelium (Johansson and Hansson, 2016). The mucus layer not only creates a physical barrier between the host and commensals but also provides an energy source for bacterial growth (Johansson et al., 2015; Desai et al., 2016). In healthy individuals, the gut microbiota is accompanied by a thicker mucus layer. In contrast, thinner mucus and gut dysbiosis have been implicated in the development of CRC through the underlying mechanism of gut microbiota and its metabolites stimulating mucus secretion (Petersson et al., 2011; Earle et al., 2015). In this review, we summarize the recent studies that focus on the role of microbiota and bacterial metabolites in mucins in CRC and offer different bacteria-targeted therapies for mucin regulation (**Figure 1**).

## 2 Role of mucins in CRC

In contrast to the small intestine (which comprises a single mucus layer), two distinct mucus layers are involved in the colon. It is composed of an outer layer exposed to commensal microbiota and an inner layer that is firmly and densely attached to the epithelium (Pothuraju et al., 2020). The inner mucus layer is rich in mucin-2 (MUC2), produced by specialized cells of the host called goblet cells, and permits less bacterial penetration into the intestinal epithelium (Desai et al., 2016). Moreover, the numerous *O*-linked glycans in the outer layer cannot only provide bacterial habitats but also serve as an energy source for bacteria (Zhang et al., 2021). The mucus layer is constantly renewed and can be rapidly adjusted to alternations in the intestinal microenvironment against bacterial invasion and activation of inflammatory responses. However, the dysfunction of the mucus layer allows the microbiota to come into contact with the intestinal epithelium, affecting the initiation and progression of CRC via initiating modifications of epithelial cells and triggering intestinal inflammation responses (Coleman et al., 2018; Yu, 2018). Hence, we focus on the mucins, including MUC2, mucin-5AC (MUC5AC), mucin-5B (MUC5B), and mucin-6 (MUC6), and systematically review their composition and function in CRC.

### 2.1 MUC2

MUC2 is the most abundant colonic mucin and forms the basis of the mucus layer. It covers the surface of intestinal mucosa in the form of gelatin (Yamashita and Melo, 2018). It is accepted that MUC2 protein plays an important role in keeping the intestinal tract healthy, while abnormal levels of MUC2 can be found in CRC patients. Numerous studies have indicated that MUC2 mucin production was reduced in patients with CRC (Bu et al., 2010; Al-Khayal et al., 2016), and higher MUC2 expression was negatively correlated with TNM stage, lymphatic metastasis, and prognosis of CRC (Elzagheid et al., 2013; Li et al., 2018). Moreover, murine models have demonstrated that MUC2^−/−^ mice allowed bacteria to contact with the intestinal epithelium, resulting in inflammation and colon cancer (Wenzel et al., 2014). An absence of MUC2 mucin expression was closely related to high methylation modification of the MUC2 promoter and a glycosylation defect of the MUC2 gene in CRC cells (Yonezawa and Sato, 1997; Biemer-Hüttmann et al., 2000).

### 2.2 MUC5AC

MUC5AC is mainly secreted by gastric goblet cells, which belong to gastric mucins. Concurrent with these studies, the expression of MUC5AC was not observed in normal colorectal epithelial cells, but its expression was significantly increasing in CRC tissues (Krishn et al., 2016). Abnormal expression of MUC5AC was related to microsatellite instability (MSI) status and poor differentiation. Moreover, MSI status was determined by MUC5AC demethylation, indicating that MUC5AC hypomethylation was a promising marker for MSI in CRC (Renaud et al., 2015). Higher MUC5 expression in CRC patients was positively associated with a high lymph node metastasis rate, poor cell differentiation, and late-stage CRC (Wang et al., 2017). *In vitro*, blocking the expression of MUC5AC in SW620 cells by siRNA technology significantly induced cell apoptosis and G1-phase cell cycle arrest and inhibited tumor cell invasion and migration (Zhu et al., 2016). The above results suggest that MUC5AC acts as a potential target for the treatment of CRC.

### 2.3 MUC5B

MUC5B is mainly expressed in the bronchus, gland, cervix, gallbladder, and pancreas and less expressed in a subset of goblet cells at the bottom of the colonic crypts in humans (Vandenhaute et al., 1997; van Klinken et al., 1998). Consistent with the mechanism of MUC5AC, overexpression of MUC5B is associated with poor outcomes in different types of gastrointestinal cancers. In HT-29 MTX cells and LS174T cells, respectively, belonging to gastric and intestinal cancer cell lines, the expression of MUC5B was significantly increased (Lesuffleur et al., 1995). To better understand the abnormal expression of the MUC5B on the pathogenesis of cancer cells, silencing the MUC5B gene in the colon cancer cell line LS174T and gastric cancer cell line KATO-III efficiently restrained cell proliferation and migration by regulating the Wnt/β-catenin pathway (Lahdaoui et al., 2017).

### 2.4 MUC6

MUC6, which is highly similar to MUC5AC, belongs to gastric mucins, but it is mainly rich in glandular epithelial cells. Lower expression of MUC6 has been reported to be associated with increased tumor cell mobility in CRC (Tsai et al., 2015). Moreover, overexpression of the MUC6 in patients with CRC had long PFS and cancer-specific survival (Betge et al., 2016). Although current studies indicate that MUC6 plays a protective in the occurrence and development of CRC, the specific mechanism needs to be verified in future experiments.

## 3 Role of gut microbiota in mucins of CRC

Despite several studies emphasizing the role of mucins in CRC, the modulating effects of gut microbiota on mucins are often ignored. Some species of pathogenic and commensal bacteria could degrade mucins or use them as attachment sites, promoting their colonization and replication. These invasive strains blinding the intestinal epithelium drive the transition to a pro-inflammatory microenvironment that accelerates colorectal tumorigenesis. Several pathogenic and commensal bacteria have been associated with CRC, including *Fusobacterium nucleatum*, *Bacteroides fragilis*, *Streptococcus gallolyticus*, *Escherichia coli*, and *Enterococcus faecalis*. Moreover, it has been shown that the influence of gut microbiota on the mucus layer of CRC requires the formation of bacterial biofilms. Here, we highlight the potential role of gut microbiota and their biofilms in regulating the mucins of CRC.

### 3.1 Fusobacterium nucleatum

The obligate anaerobic, Gram-negative bacterial species *Fusobacterium nucleatum* (*F. nucleatum*) is a normal inhabitant of the human gut and mouth. It has been recognized as an opportunistic pathogen implicated in CRC (Tahara et al., 2014; Chen et al., 2017). Recent studies have demonstrated that an abundance of *F. nucleatum* is enriched in CRC tissue in comparison to normal tissue (Kostic et al., 2012; Warren et al., 2013; Ye et al., 2017; King and Hurley, 2020). The increasing number of *F. nucleatum* in CRC patients is associated with poor survival (Mima et al., 2016). Pro-tumorigenic effects of *F. nucleatum* on CRC were associated with dysfunction of the intestinal mucosal barrier and the secretion of pro-inflammatory factors. Invasive strains of *F. nucleatum* accelerated mucin secretion, which resulted in the rapid depletion of mucin stores from goblet cells and subsequently breached the mucus layer (Dharmani et al., 2011). Meanwhile, the FadA adhesion protein secreted by *F. nucleatum* provoked the β-catenin signaling pathway in intestinal epithelial cells by interacting with E-cadherin, leading to upregulation of pro-inflammatory responses and pro-oncogenic pathways in colorectal cancer cases (Rubinstein et al., 2013; Rubinstein et al., 2019). Additionally, supplementation with *F. nucleatum* isolated from a patient with inflammatory bowel disease (IBD) in Apc^Min/+^ mice promoted tumor progression (Hooper and Macpherson, 2010; Kostic et al., 2013). Therefore, *F. nucleatum* could serve as a potential bacterial marker for the diagnosis and treatment of CRC.

### 3.2 Bacteroides fragilis

*Bacteroides fragilis* (*B. fragilis*), belonging to the *Bacteroidetes* phylum, is a common obligate anaerobic, Gram-negative gut bacterium. Although *B. fragilis* acted as a common colonic symbiote with an affinity for mucosal colonization, enterotoxigenic *B. fragilis* (ETBF), a subset of *B. fragilis* secreting a specific enterotoxin, had been shown to promote the development of CRC (Wu et al., 2009). ETBF exhibited more stable colonization in the colonic epithelial crypts of CRC and rapidly damaged the structure and function of colonic epithelial cells, such as by cleaving the tumor suppressor protein E-cadherin (Dai et al., 2019). The accumulation of ETBF strains in crypts has been shown to be essential for tumor formation via activator of transcription 3 (STAT-3) and an IL-17-dependent pro-carcinogenic inflammatory response (Wu et al., 2009; DeStefano Shields et al., 2016). Thus, ETBF has a role in triggering mucosal inflammation and promoting the carcinogenesis of colorectal cancer. Further research is needed to ascertain how the production of toxic metabolites from *B. fragilis* influences carcinogenesis by regulating the mucosal barrier.

### 3.3 Streptococcus gallolyticus

*Streptococcus gallolyticus* (*S. gallolyticus*), formerly known as *Streptococcus bovis* (*S. bovis*) biotype I, is a Gram-positive bacterium of humans belonging to the *Firmicutes* family. It acted as one of the few opportunistic pathogens that was a reported risk factor for CRC (Corredoira-Sánchez et al., 2012; Boleij and Tjalsma, 2013). Previous studies showed that an abundance of *S. gallolyticus* was enriched in CRC-mucosal tissues as compared to healthy tissue (Abdulamir et al., 2010). Another study published in 2018 found that tumor-bearing mice had an increased level (up to 1,000-fold) of *S. gallolyticus* in the gut (Aymeric et al., 2018). *S. gallolyticus* was mainly found entrapped in the mucus layer through the Pil3 pilus. Overexpression of MUC5AC in CRC could favor the adhesion of *S. gallolyticus* through Pil3 pili and thereby promotes colonization of *S. gallolyticus* (Martins et al., 2015; Martins et al., 2016). However, the role of *S. gallolyticus* in the occurrence and development of CRC is still controversial. One study within CRC patients showed that *S. gallolyticus* was more prevalent in pre-malignant tissue and drove carcinogenesis (Pasquereau-Kotula et al., 2018). On the contrary, another study revealed that *S. gallolyticus* probably only promoted tumor development after CRC had already begun (Butt et al., 2018). It should be noted that *S. gallolyticus* contributing to mucins before or after initiation of CRC certainly needs further experimental exploration.

### 3.4 Escherichia coli

*Escherichia. coli* (*E. coli*) is a Gram-negative, facultative anaerobic bacteria of the *Enterobacteriaceae* family. While *E. coli* is a gut commensal bacterium, more studies have shown that higher levels of *E. coli* were colonized in the colonic mucosa of CRC patients compared with that in healthy people (Denizot et al., 2015; Veziant et al., 2016). *E. coli* binding to the host intestinal epithelium damages the mucus layer and promotes colitis, which eventually leads to dysplasia and CRC (Martin et al., 2004; Elliott et al., 2013). *E. coli* can reduce the mucus layer and promote tumor growth due to the production of enterotoxins. *In vitro*, incubation of *E. coli* with HT-29 colon carcinoma cells resulted in reduced MUC2 glycoprotein levels via the secretion of Shiga toxins (Xue et al., 2014). Moreover, polyketide synthase (pks) island harbored by *E. coli* codes for the production of colibactin, which had been found in CRC patients and promoted colonic carcinogenesis (Cougnoux et al., 2014). Colonization with pks+ *E. coli* induced carcinogenesis via mucus damage and thereby promoted more pks+ *E. coli* binding to the intestinal epithelium, which increased colonic epithelial cell double-strand DNA breaks (Lasry et al., 2016; Dziubańska-Kusibab and Berger, 2020). Thus, genotoxic compounds from *E. coli* play a major role in promoting colorectal tumorigenesis.

### 3.5 Enterococcus faecalis

*Enterococcus faecalis* (*E. faecalis*) belongs to the *Firmicutes* and is a Gram-positive, facultatively anaerobic bacteria in humans of the gut commensal bacterium. Despite *E. faecalis* being part of normal gut flora, accumulating evidence suggests that systemic infection and CRC are closely related to the colonization of *E. faecalis*. Some studies have shown that higher *E. faecalis* levels were detected in patients with CRC compared with healthy controls (Balamurugan et al., 2008; Zhou et al., 2016). Supplementation of *E. faecalis* in the IL-10 knockout mice promoted colitis and resulted in CRC (Lucas et al., 2017). *E. faecalis* contributed to CRC pathogenesis due to its reactive oxygen species (ROS) production, which induces DNA damage and chromosomal instability in the colonic epithelium (Huycke et al., 2002). Moreover, *E. faecalis* binding mucin layers via biofilm or pilus promoted intestinal colonization and translocated through the intestine, causing systemic infection (Khan et al., 2018; Banla et al., 2019). According to evidence, *E. faecalis* may serve as biomarkers for the diagnosis and treatment of CRC with infection.

### 3.6 Bacterial biofilms

Biofilms are formed on the surface of gastric or intestinal epithelia and interact with the secreted or membrane-bound mucin, which affects mucin production. It has been reported that mucus-invasive biofilms are present in the colon of over 50% of CRC patients, whereas they are found in only 13% of healthy individuals (Dejea and Sears, 2016). Biofilms tend to invade the colonic mucus layer and present an important factor in CRC.

*E. coli* formed biofilms and used mucus as a source of energy through its digestion, which harbored its virulence genes associated with CRC (Sicard et al., 2018). *F. nucleatum* is considered to be a central player in the formation of biofilms. A clinical study has found that *F. nucleatum* and its biofilms were enriched in CRC tissues, which indicated that these bacterial species had a propensity for biofilm formation (Nakatsu et al., 2015). The increased presence of *B. fragilis* and *Enterobacteriaceae* and their ability to form biofilms could play a role in the development of CRC. Once these biofilm-positive bacteria invaded the colonic mucosal layer and came into direct contact with mucosal epithelial cells, they could cause CRC development in this population (Dejea et al., 2018). Hence, the mechanism driving the presence of tumor-associated biofilms in the mucus layer of CRC requires further investigation.

## 4 Role of bacterial metabolites in mucins of CRC

Dietary components in the large intestine are fermented by the microbial community to produce a wide range of metabolites. The major fermentation products are short-chain fatty acids, bile acids, and tryptophan, which are crucial for gut homeostasis (Feng et al., 2018). More evidence has become increasingly clear that the microbiota’s metabolic products strongly influence the intestinal mucus layer formation and development of CRC (Gill and Rowland, 2002; Schwabe and Jobin, 2013). Below, we describe the role of bacterial metabolites in regulating the mucin of CRC.

### 4.1 Short-chain fatty acids

In the metabolites of the gut microbiota, short-chain fatty acids (SCFAs) are considered the most important bacterial products. The nonabsorbable dietary fibers and resistant starches are selectively fermented by microorganisms, resulting in the production of SCFAs (butyrate, propionate, and acetate) (Koh et al., 2016). SCFAs could create a barrier between the lumen and the near-gut epithelium, leading to the activation of the MUC2 expression in the intestinal barrier and showing anti-inflammatory effects by regulating G protein-coupled receptors (Maslowski et al., 2009). Various studies have demonstrated that SCFAs also aid in improving epithelial barrier function by maintaining a good balance between intestinal immunity and inflammation (Schulthess et al., 2019; Markowiak-Kopeć and Śliżewska, 2020). In addition, SCFAs inhibited the colonization of *F. nucleatum* in patients with CRC due to shortening intestinal transit time and a change in the PH of the gut (Mehta et al., 2017). Among SCFAs, butyrate played an important role in colonic inflammation and was mainly produced by *Firmicutes*, *Eubacterium*, *Ruminococcaceae*, and *Clostridia* (Ohira et al., 2017). In clinical trials, fecal butyrate levels and butyrate-producing bacterial species were significantly decreased in patients with advanced colorectal adenoma (Chen et al., 2013). Furthermore, butyrate is thought to have a preventative impact on CRC by regulating mucin expression. A study of the effect of butyrate on mucin secretion in LS174T CRC cells indicated that butyrate could increase MUC2 levels by acetylation and methylation of histones of the MUC2 promoter (Burger-van Paassen et al., 2009). Also, treatment with butyrate in LS174T cells significantly increased mucin protein content and improved probiotic strains, thereby inhibiting the attachment of pathogenic *E. coli* (Jung et al., 2015). Above all, SCFAs, especially butyrate, are important to maintain intestinal mucus layer homeostasis and prevent CRC.

### 4.2 Bile acids

In the context of lipid metabolism, bile acids (BAs) and their derivative molecules play an important role in human metabolism. BAs are synthesized in the host liver and subsequently translated by the gut microbiota to secondary BAs (lithocholic acids and deoxycholic) in the colon (Sayin et al., 2013). A diet containing saturated fats increased the production of BAs and risk of CRC by inducing gut dysbiosis (Liu et al., 2020). In Apc^min/+^ mice, supplementation with BAs could enhance the relative abundance of *Akkermansia* and *Bacteroides* and decrease SCFAs and MUC2 expression, leading to cancer progression via activating STAT3 signaling (Wang et al., 2019). Among secondary BAs, deoxycholic acid (DCA) was considered a tumor promoter in CRC. Fecal concentrations of DCA increased the risk of CRC (Ocvirk et al., 2020), and enhanced DCAs were also found in patients with intra-mucosal carcinomas and numerous polypoid adenomas (Yachida and Mizutani, 2019). Interestingly, treatment of HM3 colon cancer cells with DCA resulted in abnormal expression of MUC2 by positive multiple pathways (Lee et al., 2010). Furthermore, pseudo-germ-free Apc^min/+^ mice induced by antibiotic streptomycin received fecal microbiota from DCA-fed animals, leading to low-grade inflammation and promoting intestinal carcinogenesis (Cao et al., 2017). Thus, BAs are positively correlated with the incidence of CRC, and understanding interactions between BAs and mucins is helpful for CRC therapy.

### 4.3 Tryptophan

Among the metabolism of amino acids supplied through food high in protein, tryptophan (Trp), which acts as a vital amino acid, plays an important role in the maintenance of inflammatory response and intestinal permeability. Some of Trp is catalyzed by host tryptophanase into kynurenine, while others are catabolized by bacteria (*Lactobacillus, Clostridium sporogenes*, etc.) to serotonin, tryptamine, and indole derivatives (indole-3-ethanol-IEt, indole-3-pyruvate-IPyA, and indole-3-aldehyde-I3A and 3-indole-propionic acid (IPA)) (Devlin et al., 2016). These indole derivatives could strengthen the mucosal layer and enhance MUC2 expression by regulating aryl hydrocarbon receptor (AhR) and pregnane X receptor (PXR) (Venkatesh et al., 2014; Gheorghe et al., 2019; Scott et al., 2020). Indole derivatives activating AhR facilitate the proliferation of epithelial cells and the expression of antimicrobial peptide and mucin production while reducing LPS-mediated inflammation (Venkatesh et al., 2014; Lanis et al., 2017; Taleb, 2019). Trp metabolites also enhanced intestinal integrity through the activation of PXR (Venkatesh et al., 2014). Moreover, supplementation with IPA in rats with a high-fat diet could repair the intestinal mucosal barrier via increased MUC2 expression (Li et al., 2021). Therefore, Trp is considered a potential target for CRC treatment, and more research is needed to fully comprehend its role in modulating mucus layer synthesis during carcinogenesis.

## 5 The potential therapy for mucins of CRC

As described previously, the gut microbiota and its metabolites play a crucial role in the intestinal mucus layer of CRC. Therefore, obtaining favorable modulations of the gut microbiome and metabolic activities to protect gut barrier function is a promising strategy for CRC prevention and treatment. The various strategies, such as probiotics, prebiotics, and fecal microbiota transplantation, are considered below.

### 5.1 Probiotics

Probiotics are live microorganisms that, when administered in adequate amounts, confer a health benefit on the host. The probiotics exert a protective effect against CRC by competing with pro-carcinogenic microbiota, modulating host immunity, and enhancing the intestinal barrier (Fong et al., 2020). On an ecological level, some probiotics could suppress the proliferation of pathogenic bacteria by secreting antimicrobial peptides. The consumption of probiotics like *Lactobacillus* and *Bifidobacterium* could reduce the abundance of *Clostridium*, *Bifidobacterium*, *Roseburia*, and *Faecalibacterium* bacteria enriched in CRC patients and inhibit the colonization of commensal bacteria such as *E. coli*, *E. faecalis*, *F. nucleatum*, and *S. gallolyticus* (Chen et al., 2019). Other probiotics may function in CRC prevention by modifying the immune response. A chemical-induced animal model study revealed that orally administered VSL#3 probiotic cocktail meliorated colitis-associated tumor development through the reduction of STAT-3 expression (Do et al., 2016). Moreover, probiotic administration could strengthen the mucosal barrier in CRC treatment. One clinical trial indicated that a combination of prebiotic inulin and two probiotic strains, *B. lactis Bb12* and *L. rhamnosus GG*, improved epithelial barrier function and reduced colorectal proliferation in patients with adenomatous or cancerous lesions (Rafter et al., 2007). Additionally, several studies have reported that probiotics reduced the frequency of severe diarrhea and abdominal discomfort in CRC patients induced by immunotherapy and chemotherapy by repairing the gut barrier (Liu et al., 2011; Demers et al., 2014). Therefore, probiotics confer health benefits to the gut barrier function of CRC.

### 5.2 Prebiotics

Prebiotics are derived from nondigestible carbohydrates in the diet, which are defined as “composition selectively fermented by microorganisms conferring host health benefits” (Gibson et al., 2017). Prebiotics are selectively utilized by host microorganisms, prompting the production of beneficial metabolites to restore intestinal homeostasis and barrier integrity (Nagpal and Yadav, 2017). Thus, consuming prebiotic-rich dietary foods that are high in fiber and low in fat and processed meat have been suggested to protect against CRC. A high-fiber diet showed a better response to prebiotics, which resulted in longer transit time in the intestine and greater immune surveillance to inhibit the mucosal colonization of invasive-adherent bacteria (Mehta et al., 2017). A recent meta-analysis study demonstrated that a high-fiber intake, particularly of whole grains and dairy products, was associated with a decreased risk of CRC (Aune et al., 2011). By contrast, western diets that were rich in red and processed meat influenced the integrity of the intestinal mucus layer, altered gut microbiota, and increased the risk of CRC (Tan and Chen, 2016).

In general, the main prebiotics included fructose-oligosaccharides (FOS) and galacto-oligosaccharides (GOS). Several studies have shown the protective effects of FOS and GOS against CRC progression via modulating gut microbiota and mucus layer function (Valcheva and Dieleman, 2016; Davani-Davari et al., 2019). FOS from nondigestible carbohydrates is absorbed by the small intestine and transferred to the colon, where they contribute to the specific stimulation of endogenous probiotics (*lactobacilli* species and *bifidobacteria*) (Tuohy et al., 2001). A study of the effects of FOS on the gut microbiotas of healthy humans revealed that FOS supplementation could increase the concentration of *bifidobacteria* in the feces, along with stabilizing neutral sterols and host bile acid content, which were involved in CRC progression (Bouhnik et al., 1996). Furthermore, feeding with FOS showed a promising increase in the relative abundance of *Lactobacillus* and *Bifidobacterium* and the intestinal mucosal barrier in rats (Lima et al., 2018). In the Apc^Min/+^ mouse model, feeding of FOS effectively inhibited the development of tumors in the colon by activating the antitumor immunity (Pierre et al., 1997). Besides influencing the microbiota, another prebiotic, GOS, is selectively degraded by the gut microbiota, leading to the production of SCFAs, which can, in turn, reduce the risk of CRC development via regulating mucus barrier functions (Ohtsuka et al., 1991; Rowland et al., 1998). *In vitro*, GOS induced increased expression of MUC2 at the transcript levels and its co-secreted molecule trefoil factor-3 in LS174T cells (Figueroa-Lozano et al., 2020). *In vivo*, GOS supplementation for 4 days resulted in higher expression of MUC2 at the transcript level in BALB/c mice (Leforestier et al., 2009). Moreover, oral administration of GOS (derived from lactulose) for 20 weeks inhibited colon tumors in the CRC rat model (Fernández et al., 2018). Overall, although the diet containing FOS and GOS provides beneficial effects on gut homeostasis, the mechanism of prebiotics on the mucus layers of CRC needs further exploration.

### 5.3 Fecal microbiota transplantation

Based on the crucial role of the intestinal microbiome in the pathogenesis of CRC, fecal microbiota transplantation (FMT) involved in bacteria-related therapies is gaining more attention. FMT refers to fecal stools from healthy donors transferred to patients *via* a nasoenteric tube or endoscope (Brandt and Aroniadis, 2013). The aim of FMT is to normalize gut dysbiosis and treat various gastrointestinal diseases, including IBD, *Clostridium difficile* infection (CDI), and irritable bowel syndrome (Smits et al., 2013; Choi and Cho, 2016). Currently, FMT is an established therapy for recurrent and refractory CDI with an over 90% success rate in clinical studies (Quraishi et al., 2017; Chen et al., 2018a). Although its application in the treatment of CRC patients is unexplored, some studies have been conducted on the use of FMT in murine models with CRC. Wild-type and germ-free mice fed with fecal samples from CRC patients prompted tumor cell proliferation compared to healthy stool-fed mice under dextran sulfate sodium salt/azoxymethane-induced colorectal tumorigenesis (Wong et al., 2017). Furthermore, fecal transplants from wild mice to laboratory mice also resisted chemically induced CRC (Rosshart et al., 2017). Thus, FMT may be a novel macrobiotic therapy for CRC, and further clinical studies are required to explore the safety and mechanisms of FMT in mucins of CRC.

## 6 Conclusion

The gut microbiota, bacterial metabolites, and host mucus layer are key players in protecting and maintaining the colon. In this review, we have outlined the profound effects of colonic microbiota and their ability to produce metabolites on the intestinal mucus layer that support colonic health and prevent CRC development (**Table 1**). Thus, studies on bacteria-targeted therapies for mucin provided many new ideas for CRC prevention and treatment. The interventions involved in prebiotics, probiotics, and FMT improve the mucus layer as a strategy for the prevention or treatment of CRC. In conclusion, a better understanding of the interplay between gut microbiota, bacterial metabolites, and the mucus barrier will shed light on novel therapeutic approaches to intestinal diseases, especially CRC.

**Table 1:** Impact of gut microbiota and bacterial metabolisms on mucins of CRC.

Impact of gut microbiota on mucins of CRC						
Gut microbiota	Pathogen or commensal organism	Target	Known effect on mucin	Model	Reference	
*Fusobacterium nucleatum*	Pathogen	MUC2	Decrease MUC2	Human	Rubinstein et al. (2019)
*Bacteroides fragilis*	Commensal	Mucin	Degrade and adhere to mucin	Cell culture	Wu et al. (2009)
*Streptococcus gallolyticus*	Pathogen	MUC5AC	Increase MUC5AC	Mouse	Martins et al. (2015)
*Escherichia coli*	Commensal	MUC2	Decrease MUC2	Cell culture	Xue et al. (2014)
*Enterococcus faecalis*	Commensal	Mucin	Adhere to mucin	Cell culture	Banla et al. (2019)
Impact of bacterial metabolisms on mucins of CRC						
Bacterial metabolisms	Dietary sources	Target	Known effect on mucin	Model	Reference	
Short-chain fatty acids	Nondigestible carbohydrates	Mucin	Promote mucin expression	Cell culture	Burger-van Paassen et al. (2009)
Bile acids	Fat	MUC2	Decrease MUC2	Mouse	Wang et al. (2019)
Tryptophan	Protein	MUC2	Increase MUC2	Mouse	Scott et al. (2020)	

## Author contributions

TC read the literature related to the topic and drafted the manuscript for publication. MG, WXY and JZ participated in searching the literature and preparing figures. JY, XT, JL, ZT and WJY participated in revising the manuscript. XW, QN and YZ participated in designing and revising the manuscript. All authors contributed to the article and approved the submitted version.
